# Supplementary material for: Multimorbidity and depression among older adults in India: Mediating role of functional and behavioural health
Source: PLoS One. 2022 Jun 7;17(6):e0269646. doi: 10.1371/journal.pone.0269646 (PMC9173646; doi:10.1371/journal.pone.0269646)
Supplement: S1 Appendix — (DOCX) [file pone.0269646.s001.docx]

**S1 Appendix**

**Table 1A** Associations between multimorbidity and depression among older adults: Logistic regression models (based on total sample)

| **Variables** | **Category** | **Model 1** | | **Model 2** | | **Model 3** | |
| --- | --- | --- | --- | --- | --- | --- | --- |
|  |  | **UOR** | **95% CI** | **AOR** | **95% CI** | **AOR** | **95% CI** |
| **Multimorbidity** | No | 1.00 |  | 1.00 |  | 1.00 |  |
|  | Yes | 1.28*** | 1.13-1.44 | 1.43*** | 1.27-1.61 | 1.27*** | 1.12-1.45 |
| ***Sociodemographic variables*** | | | | | | | |
| **Age** | Aged 60-69 |  |  | 1.00 |  | 1.00 |  |
|  | Aged 70-79 |  |  | 0.99 | 0.90-1.12 | 1.17* | 0.99-1.39 |
|  | Aged over 80 |  |  | 1.01 | 0.87-1.18 | 1.07 | 0.9-1.27 |
| **Gender** | Men |  |  | 1.00 |  | 1.00 |  |
|  | Women |  |  | 1 | 0.9-1.12 | 0.99 | 0.88-1.12 |
| **Residence** | Urban |  |  | 1.00 |  | 1.00 |  |
|  | Rural |  |  | 1.12* | 0.98-1.27 | 1.01 | 0.87-1.17 |
| **Education** | No Education |  |  | 1.00 |  | 1.00 |  |
|  | less than 5 years |  |  | 0.83* | 0.71-0.96 | 0.81* | 0.69-0.96 |
|  | 5-9 years |  |  | 0.74*** | 0.64-0.86 | 0.83* | 0.7-0.97 |
|  | 10 or more years |  |  | 0.58*** | 0.47-0.71 | 0.73** | 0.59-0.89 |
| **Marital Status** | Currently in union |  |  | 1.00 |  | 1.00 |  |
|  | Not in union |  |  | 1.29*** | 1.16-1.44 | 1.3*** | 1.17-1.46 |
| **Caste** | Others |  |  | 1.00 |  | 1.00 |  |
|  | SC/ST |  |  | 1.17* | 1.04-1.33 | 1.11 | 0.97-1.27 |
|  | OBC |  |  | 1.07 | 0.95-1.21 | 1 | 0.88-1.13 |
| **Religion** | Others |  |  | 1.00 |  | 1.00 |  |
|  | Hindu |  |  | 1.39*** | 1.18-1.63 | 1.42*** | 1.21-1.68 |
|  | Muslim |  |  | 1.36** | 1.1-1.67 | 1.49*** | 1.2-1.85 |
| **MPCE Quintile** | Rich |  |  | 1.00 |  | 1.00 |  |
|  | Middle |  |  | 1.03 | 0.89-1.18 | 1.08 | 0.96-1.21 |
|  | Poor |  |  | 1.06 | 0.95-1.18 | 1.05 | 0.91-1.22 |
| ***Functional health*** |  |  |  |  |  |  |  |
| **ADL disability** | No |  |  |  |  | 1.00 |  |
|  | Yes |  |  |  |  | 1.42*** | 1.26-1.61 |
| **IADL disability** | No |  |  |  |  | 1.00 |  |
|  | Yes |  |  |  |  | 1.24*** | 1.11-1.39 |
| **Poor sleep** | No |  |  |  |  | 1.00 |  |
|  | Yes |  |  |  |  | 2.08*** | 1.88-2.3 |
| **Pain** | No |  |  |  |  | 1.00 |  |
|  | Yes |  |  |  |  | 1.07 | 0.97-1.17 |
| **SRH** | Good |  |  |  |  | 1.00 |  |
|  | Poor |  |  |  |  | 1.66*** | 1.49-1.85 |
| ***Behavioural health*** |  |  |  |  |  |  |  |
| **Current smoker** | No |  |  |  |  | 1.00 |  |
|  | Yes |  |  |  |  | 1.17* | 1.01-1.35 |
| **Alcohol use** | No |  |  |  |  | 1.00 |  |
|  | Yes |  |  |  |  | 1.06 | 0.86-1.3 |
| **Physical inactivity** | No |  |  |  |  | 1.00 |  |
|  | Yes |  |  |  |  | 1.11* | 0.98-1.25 |
| **Obesity** | No |  |  |  |  | 1.00 |  |
|  | Yes |  |  |  |  | 1.56** | 1.18-2.07 |
| ***Pseudo R^2^*** |  | ***0.02*** | | ***0.018*** | | ***0.043*** | |

*%: Percentage;UOR: Unadjusted Odds Ratio; AOR: Adjusted Odds Ratio; CI: Confidence interval; Pseudo R^2^: Measure of model fitting on the same data, predicting the same outcome.*

*Model 1: Unadjusted model*

*Model 2: Adjusted for age, gender, marital status, residence, education level, religion, caste, MPCE quintile.*

*Model 3: Adjusted for Model 2, ADL disability, IADL disability, poor sleep, pain, SRH, currently smoking, alcohol use, Physical Inactivity, and obesity.*

**p < 0.05; **p < 0.005; ***p < 0.001*

**Table 1B** Associations between multimorbidity and depression among older adults: Logistic regression models (men)

| **Variables** | **Category** | **Model 1** | | **Model 2** | | **Model 3** | |
| --- | --- | --- | --- | --- | --- | --- | --- |
|  |  | **UOR** | **95% CI** | **AOR** | **95% CI** | **AOR** | **95% CI** |
| **Multimorbidity** | No | 1.00 |  | 1.00 |  | 1.00 |  |
|  | Yes | 1.35*** | 1.15-1.58 | 1.54*** | 1.30-1.82 | 1.40*** | 1.15-1.69 |
| ***Sociodemographic variables*** | | |  |  |  |  |  |
| **Age** | Aged 60-69 | |  | 1.00 |  | 1.00 |  |
|  | Aged 70-79 | |  | 1.05 | 0.81-1.29 | 1.31* | 1.02-1.69 |
|  | Aged over 80 | |  | 0.98 | 0.78-1.24 | 1.15 | 0.89-1.5 |
| **Residence** | Urban |  |  | 1.00 |  | 1.00 |  |
|  | Rural |  |  | 0.99 | 0.83-1.18 | 0.91 | 0.73-1.12 |
| **Education** | No Education | |  | 1.00 |  | 1.00 |  |
|  | less than 5 years | |  | 0.93 | 0.76-1.14 | 0.91 | 0.73-1.12 |
|  | 5-9 years |  |  | 0.79** | 0.67-0.94 | 0.85* | 0.7-1.02 |
|  | 10 or more years | |  | 0.53*** | 0.43-0.66 | 0.63*** | 0.49-0.8 |
| **Marital Status** | Currently in union | |  | 1.00 |  | 1.00 |  |
|  | Not in union | |  | 1.31** | 1.12-1.53 | 1.35*** | 1.14-1.61 |
| **Caste** | Others |  |  | 1.00 |  | 1.00 |  |
|  | SC/ST |  |  | 1.26* | 1.05-1.51 | 1.13 | 0.93-1.37 |
|  | OBC |  |  | 1.07 | 0.91-1.26 | 0.99 | 0.82-1.18 |
| **Religion** | Others |  |  | 1.00 |  | 1.00 |  |
|  | Hindu |  |  | 1.80*** | 1.42-2.29 | 1.77** | 1.38-2.28 |
|  | Muslim |  |  | 1.66** | 1.22-2.27 | 1.76*** | 1.27-2.45 |
| **MPCE Quintile** | Rich |  |  | 1.00 |  | 1.00 |  |
|  | Middle |  |  | 1.01 | 0.87-1.17 | 1.07 | 0.91-1.25 |
|  | Poor |  |  | 1.04 | 0.85-1.28 | 1.12 | 0.89-1.41 |
| ***Functional health*** | |  |  |  |  |  |  |
| **ADL disability** | No |  |  |  |  | 1.00 |  |
|  | Yes |  |  |  |  | 1.32** | 1.09-1.6 |
| **IADL disability** | No |  |  |  |  | 1.00 |  |
|  | Yes |  |  |  |  | 1.26** | 1.08-1.47 |
| **Poor sleep** | No |  |  |  |  | 1.00 |  |
|  | Yes |  |  |  |  | 1.97*** | 1.7-2.28 |
| **Pain** | No |  |  |  |  | 1.00 |  |
|  | Yes |  |  |  |  | 1.04 | 0.87-1.16 |
| **SRH** | Good |  |  |  |  | 1.00 |  |
|  | Poor |  |  |  |  | 1.54*** | 1.31-1.8 |
| ***Behavioural health*** | |  |  |  |  |  |  |
| **Current smoker** | No |  |  |  |  | 1.00 |  |
|  | Yes |  |  |  |  | 1.16* | 0.99-1.37 |
| **Alcohol use** | No |  |  |  |  | 1.00 |  |
|  | Yes |  |  |  |  | 1.07 | 0.86-1.35 |
| **Physical inactivity** | No |  |  |  |  | 1.00 |  |
|  | Yes |  |  |  |  | 1.01 | 0.85-1.17 |
| **Obesity** | No |  |  |  |  | 1.00 |  |
|  | Yes |  |  |  |  | 2.29*** | 1.48-3.53 |
| ***Pseudo R2*** | | ***0.003*** | | ***0.020*** | | ***0.043*** | |

*%: Percentage;UOR: Unadjusted Odds Ratio; AOR: Adjusted Odds Ratio; CI: Confidence interval; Pseudo R^2^: Measure of model fitting on the same data, predicting the same outcome.*

*Model 1: Unadjusted model*

*Model 2: Adjusted for age, marital status, residence, education level, religion, caste, MPCE quintile.*

*Model 3: Adjusted for Model 2, ADL disability, IADL disability, poor sleep, pain, SRH, currently smoking, alcohol use, Physical Inactivity, and obesity.*

**p < 0.05; **p < 0.005; ***p < 0.001*

**Table 1C** Associations between multimorbidity and depression among older adults: Logistic regression models (women)

| **Variables** | **Category** | **Model 1** | | **Model 2** | | **Model 5** | |
| --- | --- | --- | --- | --- | --- | --- | --- |
|  |  | **UOR** | **95% CI** | **AOR** | **95% CI** | **AOR** | **95% CI** |
| **Multimorbidity** | No | 1.00 |  | 1.00 |  | 1.00 |  |
|  | Yes | 1.21*** | 1.01-1.44 | 1.36*** | 1.16-1.59 | 1.19* | 1.00-1.41 |
| ***Sociodemographic variables*** | | |  |  |  |  |  |
| **Age** | Aged 60-69 | |  | 1.00 |  | 1.00 |  |
|  | Aged 70-79 | |  | 0.96 | 0.79-1.18 | 1.06 | 0.84-1.33 |
|  | Aged over 80 | |  | 0.99 | 0.81-1.22 | 0.98 | 0.79-1.22 |
| **Residence** | Urban |  |  | 1.00 |  | 1.00 |  |
|  | Rural |  |  | 1.21* | 1.01-1.46 | 1.11 | 0.91-1.35 |
| **Education** | No Education | |  | 1.00 |  | 1.00 |  |
|  | less than 5 years | |  | 0.72** | 0.58-0.9 | 0.72** | 0.57-0.91 |
|  | 5-9 years |  |  | 0.68** | 0.52-0.9 | 0.82 | 0.62-1.08 |
|  | 10 or more years | |  | 0.71 | 0.43-1.17 | 0.99 | 0.66-1.52 |
| **Marital Status** | Currently in union | |  | 1.00 |  | 1.00 |  |
|  | Not in union | |  | 1.30*** | 1.13-1.5 | 1.29** | 1.11-1.49 |
| **Caste** | Others |  |  | 1.00 |  | 1.00 |  |
|  | SC/ST |  |  | 1.11 | 0.92-1.30 | 1.08 | 0.9-1.3 |
|  | OBC |  |  | 1.06 | 0.89-1.26 | 1.01 | 0.85-1.19 |
| **Religion** | Others |  |  | 1.00 |  | 1.00 |  |
|  | Hindu |  |  | 1.17 | 0.94-1.45 | 1.22 | 0.98-1.52 |
|  | Muslim |  |  | 1.19 | 0.9-1.58 | 1.33 | 1-1.79 |
| **MPCE Quintile** | Rich |  |  | 1.00 |  | 1.00 |  |
|  | Middle |  |  | 1.09 | 0.94-1.27 | 1.08 | 0.92-1.27 |
|  | Poor |  |  | 1.01 | 0.84-1.21 | 1.02 | 0.84-1.19 |
| ***Functional health*** | |  |  |  |  |  |  |
| **ADL disability** | No |  |  |  |  | 1.00 |  |
|  | Yes |  |  |  |  | 1.50*** | 1.27-1.75 |
| **IADL disability** | No |  |  |  |  | 1.00 |  |
|  | Yes |  |  |  |  | 1.24** | 1.06-1.45 |
| **Poor sleep** | No |  |  |  |  | 1.00 |  |
|  | Yes |  |  |  |  | 2.17*** | 1.91-2.48 |
| **Pain** | No |  |  |  |  | 1.00 |  |
|  | Yes |  |  |  |  | 1.12 | 0.99-1.27 |
| **SRH** | Good |  |  |  |  | 1.00 |  |
|  | Poor |  |  |  |  | 1.75*** | 1.52-2.02 |
| ***Behavioural health*** | |  |  |  |  |  |  |
| **Current smoker** | No |  |  |  |  | 1.00 |  |
|  | Yes |  |  |  |  | 1.06 | 0.82-1.35 |
| **Alcohol use** | No |  |  |  |  | 1.00 |  |
|  | Yes |  |  |  |  | 0.9 | 0.64-1.28 |
| **Physical inactivity** | No |  |  |  |  | 1.00 |  |
|  | Yes |  |  |  |  | 1.23* | 1.03-1.48 |
| **Obesity** | No |  |  |  |  | 1.00 |  |
|  | Yes |  |  |  |  | 1.44** | 1.02-2.03 |
| ***Pseudo R2*** | | ***0.001*** | | ***0.015*** | | ***0.045*** | |

*%: Percentage;UOR: Unadjusted Odds Ratio; AOR: Adjusted Odds Ratio; CI: Confidence interval; Pseudo R^2^: Measure of model fitting on the same data, predicting the same outcome.*

*Model 1: Unadjusted model*

*Model 2: Adjusted for age, marital status, residence, education level, religion, caste, MPCE quintile.*

*Model 3: Adjusted for Model 2, ADL disability, IADL disability, poor sleep, pain, SRH, currently smoking, alcohol use, Physical Inactivity, and obesity.*

**p < 0.05; **p < 0.005; ***p < 0.001*
