# Supplementary material for: Multimorbidity and depression among older adults in India: Mediating role of functional and behavioural health
Source: PLoS One. 2022 Jun 7;17(6):e0269646. doi: 10.1371/journal.pone.0269646 (PMC9173646; doi:10.1371/journal.pone.0269646)
Supplement: Attachment — Submitted filename: Response to reviewers.docx [file pone.0269646.s002.docx]

**Comments to the Author**

We are grateful to have been given the opportunity to submit a revised draft of our manuscript entitled “Multimorbidity and depression among older adults in India: Mediating role of functional and behavioural health” to Plos One. We appreciate the time and effort that the editor and reviewers have dedicated to providing feedback on our manuscript. We have revised the manuscript incorporating suggestions proposed by the reviewers, which has greatly contributed to improving the manuscript. The comments and concerns that were raised by the reviewers were addressed and are highlighted in the revised manuscript. A point-by-point response to the raised comments and concerns is given below.

Reviewer #1: The research topic is interesting. Use of LASI is the major strength of the study. However, there are few concerns:

1. Were survey weights used in the analysis?

**Response – Yes, We have mentioned this in methodology section.**

2. The study highlights both direct and indirect pathways between multimorbidity and depression. However, merging healthy individual with individuals with single morbidity is not reassuring, it might have reflected poorly on the study estimates. If possible, try using mm as a categorical variable (with three distinct category).

**Response –**Dear reviewer, thanks for the kind words and acknowledging the importance of the theme of our manuscript. Even though, previous literature has shown that living with multiple health conditions is even more impactful than any one health condition, particularly in later life. Due to treatment burden and poor disease management, mental health disorders, particularly depression, are more prevalent in persons with an increasing number of physical disorders. We agree with the reviewer, but taking mm as a category variable was beyond the scope of our analysis in broader approach, but would be useful in future research.

3. The authors are requested to use standard terminology. Use of word femen is not a standard generally used in research articles.

**Response – Thank you for noticing. Comments are incorporated throughout the manuscript.**

4. Try to add more background on the point of gender differences, as it is highlighted as the major research objective in the discussion section.

**Response – Thank you for your suggestion, we have added few more points on gender differences.**

5. Authors have not properly reviewed the literature, Sanghamitra Pati and GK Mini are the two authors which needs to be cited in any article published on multimorbidity in India. Thus might reflect poorly to the international readers. Try citing articles written by researchers outside IIPS to add more weightage to your study.

**Response – Thank you for suggestions, we have incorporated your suggestions.**

6. Abstract uses the term univariate logistic regression model. What does that supposed to mean. For regression analysis it should be binary. If this statement means anything else, than consider reframing the sentence to sound more clear.

**Response – Dear reviewer, thanks for guiding. Yes, the statement is modified.**

The article can be accepted from my end.

Reviewer #2: The study “Multimorbidity and depression among older adults in India: Mediating role of functional and behavioural health” is generally well developed. It examined the influence of multimorbidity on elders’ depression and its gender disparities while assessing functional health’s and behavioral health’s mediating effects. Finally, the study revealed that multimorbid older people are more likely to suffer depressive symptoms, and the association may be mediated by certain functional health factors, especially in older women. Below are some comments for the authors’ consideration.

In the Introduction section:
1. Some hypothesis that should be required to further verified and proved by the study were stated in affirmative tone, which is obviously inappropriate. For instance, lines 90 to 91 “It is also one of the major predictors of mental health of older population.”

**Response – Thank you for suggestion. We modified the statement accordingly.**

2. It has been well told functional health could be a mediating factor between multimorbidity and depression, so it is necessary to inform that why behavioral health could also be a mediating factor. The statement in lines 99 to 101, page 4 is not sufficient.

**Response – Thank you for suggestion, we have added more information on behavioural health part.**

3. The covariates, selected in Figure 1, should be supported fully by theoretical or empirical evidence. For example, some specific indicators of covariates and functional health, such as education, income, ADL, IADL, etc., are not mentioned in the introduction section. So, it is recommended to supplement more evidences on those items to build the theoretical model.

**Response – Thank you for your suggestion. We incorporated the suggestion for ADL and IADL in the revised manuscript. As this study mainly focused on the multimorbidity and depression status considering the mediating role functional health among older adults, we have tried to cover the existing literature focusing on these aspects. While education, income and other covariates are controlled in our study which are not main focus of the study. These covariates were included in the model to ensure that these variables did not confound any of the assessed direct and indirect associations between multimorbidity status and depression. Thus, we have intentionally skipped the discussion on covariates. A separate study can be caried out to highlight mentioned factors role in multimorbidity and depression.**

4. Besides, to highlight that “Age-related multimorbidity is an escalating issue that poses a major challenge for health care systems worldwide” fully, it is better to add some evidences from developed countries, but not only from low-middle income countries.

**Response – Evidences from developed countries added in the manuscript.**

In the Methods section:
5. The activities of ADL and IADL are counted six and seven respectively, in lines 199 -205, on page 8, while it is said “a series of five and six different questions” in lines 198 -199. It is not consistent.

**Response – Thank you for noticing. Yes, it is modified now.**

6. It is not clear for the code of some variables. For example, Is never and rarely sleep coded as 0, occasionally and frequently sleep coded as 1? Pain and Self-rated health are also the same. It is recommended to introduce variables’ coding way clearly.

**Response – Dear reviewer, thank you for suggestions. Now, coding for concerning variables has been introduced clearly in the revised manuscript.**

In the Results section:
7. The statistical difference among the subgroups of various factors should be reported in Table 1.

**Response – The main objective of the Table 1 was to give the sample characteristics for the study population. Thus, the table only showed the percentage distribution of the total sample included in the study. As you suggested, we modified table 1 showing the statistical difference by gender for various factors.**

8. It is better to add statement for the meaning of “UOR, AOR, 95%CI, and Pseudo R2” clearly below the table 2.

**Response – Thanks for suggestions. Comments are incorporated.**

9. It is suggested to add the results about covariates in each model in table 2, 3, 4, and 5, which will be helpful for showing the impact of demographic characteristics on elders’ depression.

**Response – Thank you for your suggestion. Table 2 highlighted the associations between multimorbidity and depression stratified for gender. Although there are several literatures focusing range of covariates of depression, we only aimed to examine the association multimorbidity and depression controlling covariates in Table 2, further adding the gender dimension. As you suggested and for the readers clarity, we have added these tables separately in the supplementary section.**

10. It is recommended to list the indirect effect value, direct effect value and total effect value in table 3, 4 and 5, and add a note to explain the calculation formula of these effect values below the table 3.

**Response – Thank you for your suggestion. We had already given total, direct and indirect effect value in form of odds ratio. Further, the we have added a note to explain the calculation formula of these effect values in method section.**

11. It will be better to elaborate the results in tables 4 and 5 separately, and the lines 365-366, on page 16.

**Response – Comments are incorporated.**

12. There were some confused issues. Why the mediating % in table 4 and table 5 do not match the data in the text (lines 384-385, lines 387-389, on page 17)? Why is there still a mediating % of physical inactivity shown in table 5, but the text stated that physical inactivity isn’t a mediator between multimorbidity and depression for older women. (lines 389-390, on page 18).

**Response – Thank you for your observations. Now, the value of mediating percentage has been incorporated in the text according to the tables.**

13. Does the different mediating % reflect distinct impact degrees of different factors on the association between multimorbidity and depression for elders? However, the paper only stated the mediating % without further comparison, it feels leaving out more valuable information.

**Response – Comments are incorporated. Comparisons of mediating percentage among functional and behavioral health has been added in the result section as well as discussion part.**

In the Discussion section:
14. From the tables in results section, the mediating % of poor sleep in the overall sample and male sample are both the second, so, how to judge poor sleep was the strongest mediator of the examined factor in the study (lines 412- 413, on page 13)? Which is the same in lines 422 to 424, on page 13. The mediating % from the total sample and male sample show that self-rated health are both the largest, so, why is self-rated health the second stronger mediator among the variables used in the study?

**Response – Thank you for noticing. Yes, self-rated health is the strongest mediators in the possible multimorbidity-depression association. We modified both the statements in discussion part accordingly.**

15. The sentence “Poor sleep, multimorbidity,…that influences one another.” in lines 418-419, on page 13 is too affirmative, for the study did not explore the bidirectional effect between factors.

**Response – Thank you for your observations. Poor sleep, multimorbidity, and depressive symptoms all might have a bidirectional or reciprocal link that influences one another. However, further longitudinal study is required to make any causal inference. We also added this in the write up.**

16. What does the“dis”in line 431, on page 19 refer to?

**Response – The typo has been corrected.**

17. Grammar error in line 432, on page 19 “leading leads to poor quality of life”.

**Response – The error has been resolved.**

18. “the need for healthcare facilities…depression in older adults.” in lines 433- 435, on page 19, this explanation is too general and abstract to make it difficult to understand.

**Response – As you suggested. Corrections have been done in this section.**

19. The discussion section mentions the results repeatedly, but doesn’t explain the deep reasons behind the results. So, the reasons of the results are not fully discussed.

**Response – We have addressed the concerns in discussion section.**

In the Conclusions section:
20. The recommendations for prevention and treatment of chronic diseases and depression among elders should be further supplemented from perspective of gender disparities.

**Response – Thank you for your observation. As suggested, we further highlighted the gender disparities in the conclusion part.**

6. PLOS authors have the option to publish the peer review history of their article ([what does this mean?](https://journals.plos.org/plosone/s/editorial-and-peer-review-process#loc-peer-review-history)). If published, this will include your full peer review and any attached files.


If you choose “no”, your identity will remain anonymous but your review may still be made public.


**Do you want your identity to be public for this peer review?** For information about this choice, including consent withdrawal, please see our [Privacy Policy](https://www.plos.org/privacy-policy).

Reviewer #1: **Yes:** Parul Puri

Reviewer #2: No
