# Supplementary material for: Multimorbidity and depression among older adults in India: Mediating role of functional and behavioural health
Source: PLoS One. 2022 Jun 7;17(6):e0269646. doi: 10.1371/journal.pone.0269646 (PMC9173646; doi:10.1371/journal.pone.0269646)
Supplement: Attachment — Submitted filename: Response to reviewers.docx [file pone.0269646.s003.docx]

**Rebuttal Letter**

Dear Editor

Thank you for your consideration. The reviewers have raised valid comments and we have incorporated those comments which have improved our manuscript. We have also prepared a point by point rebuttal to the comments raised by the reviewers.

Much regards

**Comments to the Author**

Reviewer #1: Thank you for the opportunity to review this interesting piece of work. The authors have done a great job in addressing the comments. There are only minor issues remaining in the paper, this includes grammatical, English language errors and references sections. In my opinion, this can be addressed in the proof-reading stage. I would recommend this article to be published after a rigorous proof-reading.

Reviewer #2: Overall, I think it is a good job for revising the manuscript and responding to previous comments. While I still have a few minor comments about this manuscript:
1. In the abstract, it seems to ignore mentioning the mediating proportion of the factor “pain”.

**Response – Thank you for your comment, we have updated the abstract accordingly.**

2. Why the mediating % of physical inactivity in table 3 does not match the data in the text (lines 402-404)?

**Response – We have corrected this error in the manuscript.**

3. In lines 471-473 of discussion section, the “a third factor” is confusing.

**Response – Thank you for pointing out, we have corrected the sentence.**
